# The role of negative affect, depression, and perceived disease susceptibility in self‐reported dementia worry

**DOI:** 10.1002/alz.090612

**Published:** 2025-01-03

**Authors:** Jacklyn Gehling, Claudia Jacova, Frank Robertson, Ravneet K. Dhaliwal

**Affiliations:** ^1^ Pacific University, Hillsboro, OR USA

## Abstract

**Background:**

Worry about developing Alzheimer’s disease and related dementias (ADRD), otherwise known as dementia worry, is a construct of significant interest in ADRD‐related health behaviors. In the present analysis, we aimed to understand dementia worry in the framework of the Health Belief Model by investigating the contribution of perceived risk/susceptibility and psychological factors including depression and negative affect.

**Method:**

An English‐speaking community sample free of neurocognitive diagnoses (*n* = 279) who were aged 60‐96 (*M* = 73.8), 52% women, and had a mean education of 14.9 years (range = 10‐20) completed a Qualtrics online survey probing their perceptions of ADRD. Participants rated their worry about developing ADRD on a 5‐point scale (1 = not at all worried to 5 = very worried) and indicated which of 10 evidence‐based risk factors they believed contributed. Endorsed risk factors were summed for analysis. Participants also completed the Patient Health Questionnaire (PHQ‐2) and the Positive and Negative Affect Schedule (PANAS). A three‐step hierarchical regression analysis with age and education entered in step 1, ADRD Risk Sum entered in step 2, and PHQ‐2 and PANAS‐Negative Affect scores entered in step 3 was conducted predicting ADRD Worry. We first tested depression and negative affect in separate models in step 3 and found that each contributed. We then combined them into a single model to better understand the contribution of each.

**Result:**

Participants had a mean score of .51 on the PHQ‐2 (*SD* = 1.07), a mean score of 7.11 on PANAS‐Negative Affect (*SD* = 3.26), endorsed on average 2.77 ADRD risk factors (*SD* = 1.83), and ranked their worry at 2.05 on average (*SD* = .93). In the final model, age and education did not predict ADRD Worry, whereas ADRD Risk Sum did (R‐squared change = .03, B = .09, *p*<.05), and negative affect and depression scores contributed further (R‐squared change = .04), but only PHQ‐2 scores were predictive (B = .16, *p*<.05). The contribution of ADRD Risk Sum remained significant after entry of the affective variables in step 3.

**Conclusion:**

We found that in a community sample of adults aged 60 and above, higher levels of dementia worry were associated with higher perceived disease susceptibility and poorer emotional status indexed by negative affect and, more importantly, depression.

